# Switching in the expression pattern of actin isoforms marks the onset of contractility and distinct mechanodynamic behavior during cardiomyocyte differentiation

**DOI:** 10.14814/phy2.15171

**Published:** 2022-02-14

**Authors:** Ricardo H. Pires, Tung H. Dau, Emmanuel Manu, Nithya Shree, Oliver Otto

**Affiliations:** ^1^ ZIK‐HIKE ‐ Zentrum für Innovationskompetenz: Humorale Immunreaktionen bei kardiovaskulären Erkrankungen Universität Greifswald Greifswald Germany; ^2^ DZHK ‐ Deutsches Zentrum für Herzkreislaufforschung Greifswald Germany; ^3^ FLI ‐ Friedrich‐Loeffler‐Institut Greifswald Insel Riems Germany

**Keywords:** cardiomyocyte, cell mechanics, differentiation, stem cells

## Abstract

Differentiation of cardiac progenitor cells (CPC) into cardiomyocytes is a fundamental step in cardiogenesis, which is marked by changes in gene expression responsible for remodeling of the cytoskeleton and in altering the mechanical properties of cells. Here we have induced the differentiation of CPC derived from human pluripotent stem cells into immature cardiomyocytes (iCM) which we compare with more differentiated cardiomyocytes (mCM). Using atomic force microscopy and real‐time deformability cytometry, we describe the mechanodynamic changes that occur during the differentiation process and link our findings to protein expression data of cytoskeletal proteins. Increased levels of cardiac‐specific markers as well as evolution of cytoskeletal morphology and contractility parameters correlated with the expected extent of cell differentiation that was accompanied by hypertrophic growth of cells. These changes were associated with switching in the balance of the different actin isoforms where β‐actin is predominantly found in CPC, smooth muscle α‐actin is dominant in iCM cells and sarcomeric α‐actin is found in significantly higher levels in mCM. We link these cytoskeletal changes to differences in mechano‐dynamic behavior of cells that translate to changes in Young's modulus that depend on the cell adherence. Our results demonstrate that the intracellular balance of actin isoform expression can be used as a sensitive ruler to determine the stage of differentiation during early phases of cardiomyocyte differentiation that correlates with an increased expression of sarcomeric proteins and is accompanied by changes in cellular elasticity.

## Graphical abstract

The evolution of the mechanical properties of cardiomyocytes during development has been investigated in dependence of the expression of cytoskeletal proteins. We found that at early stages of differentiation, cells possess a Young's modulus that strongly depends on their state of adherence. As cells approach maturation, where sarcomere assembly becomes evident, we observe an increased contractile activity that is accompanied by increased elasticity.

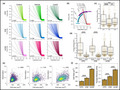

## 1 INTRODUCTION

The myocardium of adult humans shows a very low potential for self‐regeneration, with cardiomyocytes undergoing turnover rates estimated at 0.5%–1% yearly (Bergmann et al., 2015). Thus, in the event of myocardial injury, damaged cardiomyocytes are typically replaced by cardiac fibroblasts that increase the risk of heart failure (González et al., 2018, 2019). But the heart appears to possess more subtle compensatory mechanisms that only in the last decade have become clearer. These may rely on the *in situ* differentiation of stem cells, or in dedifferentiation of cardiomyocytes back to a more immature state, followed by proliferation and redifferentiation. Both mechanisms hold therapeutic promise, either through the transplantation of engineered or embryonic stem cells, or through the administration of paracrine factors that induce cardiomyocyte self‐renewal. Thus, the transitions in the state of differentiation and maturation in cardiomyocytes are not only relevant from a developmental standpoint, but they are also a window to reparatory mechanisms that may be harnessed for therapeutic purposes.

It is now well established that cellular mechanics is a sensitive biomarker for many physiological changes occurring as part of normal and aberrant development. In cardiomyocytes, an extensive number of studies have highlighted the relevance of cellular mechanosensing as an important gateway to promote cardiomyocyte differentiation and maturation (Castillo et al., 2020). These processes have been linked to changes in elasticity of both cells (Tan et al., 2012) and heart tissue (Jacot et al., 2010) and have been proposed to stem from the cytoskeletal remodeling that accompanies differentiation and progresses through maturation of cardiomyocytes (Ali et al., 2020). While it is challenging to dissect the contribution of individual cytoskeletal elements toward global cellular mechanical properties, it is generally accepted that both microtubules and the filamentous actin network are major contributors to cellular viscoelasticity.

The expression pattern of actin isoforms is strongly modulated along the cardiac lineage, and cardiomyocyte differentiation has been shown to entail the sequential activation of actin genes in both avian embryos (Ruzicka & Schwartz, 1988) and human stem cells (Clément et al., 2007). In both cases, initial differentiation events are associated with the transient expression of smooth‐muscle α‐actin that is later replaced by both cardiac and skeletal isoforms. By contrast, β‐actin has been reported to be downregulated in developing avian hearts (Lin & Redies, 2012), although transcriptomic analysis indicated the opposite during stem cell differentiation (Cao et al., 2008). Similar findings have also been reported for skeletal myocytes (Bains et al., 1984), suggesting that this pattern of modulating actin isoform expression may be a feature of the development of striated muscles. Given that the different isoforms are not, for the most part, functionally equivalent, their regulation at the gene level effects the cytoskeletal architecture.

Compared with the changes in the actin network, cardiomyocyte differentiation appears to impact microtubule assembly in a more subtle fashion. Microtubules, which are composed of α‐ and β‐tubulin dimers, are an important scaffold structure for intracellular cargo delivery via kinesins and dyneins. In proliferating cells, they are central elements in the assembly of the mitotic spindle. But despite the reduced proliferative capabilities of cardiomyocytes, microtubules hold an important mechanical role during contraction (Caporizzo et al., 2020), and several microtubule‐related dysfunctions have been linked to cardiomyopathies (Li et al., 2020). During differentiation, the transition of microtubules to acentrosomal juxtanuclear sites is a hallmark of a post‐mitotic state and appears to enhance the cardiomyocyte phenotype (Ng et al., 2020). Regulation of the expression of tubulins during differentiation is more often noted with respect to the γ‐isoform, whereas α‐ and β‐tubulins appear to be more finely regulated by post‐translational modifications (Becker et al., 2020).

While differentiation entails changes in the cellular cytoskeleton that have been well noted, its impact in the mechanodynamc properties of cells in the cardiac lineage is more often overlooked. An earlier study found that the epicardial surface of ventricles shows an increase in elasticity from embryonic to neonatal stages (Jacot et al., 2010). However, it is also known that the dense collagenous extracellular matrix in the cardiac tissue is an important contributor to the mechanical properties of the heart (Hochman‐Mendez et al., 2020; Perea‐Gil et al., 2015). Therefore, the significance of the cytoskeletal remodeling that occurs during early stages of differentiation toward the mechanical properties of those cells remains to be elucidated.

Here, we have used a combination of techniques to assert the extent of maturation of cardiac progenitor cells (CPC) which we differentiated into immature cardiomyocytes (iCM), and further compared with cardiomyocytes that display a more differentiated phenotype (mCM). We observed that early stages in cardiomyocyte differentiation are marked by a rise in the levels of well‐known cardiac‐specific markers which increased with the extent of differentiation. In turn, development of contractile activity could be linked to higher levels of sarcomere organization, as well as switching of actin expression profile. To gain further insight into the process of cytoskeletal remodeling, we used colloidal force spectroscopy and real‐time deformability cytometry (RT‐DC) to mechanically probe the cells and found that more differentiated cells appeared larger and exhibited differences in mechanical properties that depend on their adherent state. Our work provides evidence that even at early stages of development, when the cardiomyocyte phenotype is not yet fully amplified, significant changes in the material properties of cells can be detected. These changes could be correlated with major phenotypic markers of cardiomyocyte development, thus supporting the view that cellular mechanics can be used as a sensitive label‐free marker to follow differentiation along the cardiac lineage.

## 2 MATERIALS AND METHODS

### 2.1 Cell culture

Cardiac progenitor cells, iCM, and mCM were cultured in surfaces coated with 200 µl/cm^2^ of basement membrane matrix solution (Geltrex^TM^, #A1569601; ThermoFisher Scientific) for 2 h at 37°C. Long‐term cell culture occurred in a basic maintenance medium comprised of William's E medium (#A1217601; ThermoFisher Scientific) supplemented with cocktail B of the primary hepatocyte maintenance supplements pack (#CM4000; ThermoFischer Scientific) and 25 µg/ml of gentamicin (#15750037; ThermoFisher Scientific). Additional supplements were introduced in the medium depending on the requirements of each culture protocol (see below).

CPC (iCell Cardiac Progenitor Cells, #R1093; Fujifilm Cellular Dynamics) were derived from human pluripotent stem cells (hiPSC) and obtained based on the procedure reported by Ma et al. (2011) and cryopreserved 8 days after the start of differentiation. Following the manufacturer's instructions, upon thawing the cell suspension vials, cells were cultured for 2 days in the presence of 1 µg/ml of recombinant human basic fibroblast growth factor (bFGF, #50361; Biomol).

Differentiation of CPC into iCM was done also observing manufacturer's instructions by supplementing the basic maintenance medium with 10 µM XAV939 (#X3004; Sigma‐Aldrich) and 2.5 µM SB431542 (#S4317; Sigma‐Aldrich) during the first 48 h after seeding CPC (Drowley et al., 2016). Thereafter, cells were cultured only in the basic maintenance medium for a total of 7 days.

mCM (iCell Cardiomyocytes^2^, # R1017; Fujifilm Cellular Dynamics) were derived from hiPSC and obtained based on the procedure reported by Ma et al. (2011) and cryopreserved 30−32 days after the start of differentiation. Once thawed, cells were seeded using iCell Cardiomyocytes Platting Medium (#M1001; Fujifilm Cellular Dynamics) and left to adhere to the culture surface for the first 4 h. The medium was then exchanged for the basic maintenance medium and cells cultured in this medium for a total of 7 days.

### 2.2 Cardiomyocyte contractility analysis

#### 2.2.1 Sample preparation

All cell types were seeded in imaging chambers (µ‐Slide eight well, #80826; Ibidi, GmbH) and cultured as described above and left to acclimate for 30 min to the setup conditions prior to measurements.

#### 2.2.2 Data acquisition

Recording of cell motion using phase‐contrast video microscopy was done on an Eclipse Ti microscope (Nikon) controlled by NIS‐Elements AR (v3.22) software (Nikon). Detection was implemented using an iXon+897 EMCCD camera (Andor) as 14‐bit images. Atmosphere control was achieved using INUB‐GSI stage top incubator (Tokai Hit) set to 37°C and a GM‐4000 Gas Mixer (Tokai Hit) set to 5% CO_2_ and 160 ml/min. For every replicate, recording was set over a period of 120 s at a frame rate of 25 frames per second.

#### 2.2.3 Data analysis

Analysis of contractility by video microscopy data was performed as reported earlier by us (Pires et al., 2019) and is based on the method of difference between consecutive frames (Migliore et al., 2006; Singla, 2014; Tan et al., 2018). Thus, for a recorded video comprised of *n* frames, the following operation is performed:

$$\Delta I\left(x,y\right)=\left|I_{k}\left(x,y\right)‐I_{k‐1}\left(x,y\right)\right|$$

in which Δ*I*(*x*,*y*) is the absolute difference in grayscale intensity for every (*x*,*y*) pixel position, between any *k* frame (*I_k_*) and the previous frame (*I_k_*_−1_). The result of this operation is a video containing *n*−1 frames where the difference (Δ) information is stored for every pixel position. The mean pixel intensity across each Δ frame was then determined, which resulted in a trace where periods of contractile activity are distinguishable from quiescent phases. For a developed cardiomyocyte phenotype, motion‐rich periods are characterized by peak‐doublets, with the first peak resulting from cell contraction, and the second associated with the relaxation phase. For a given trace, the amplitude of such motion events is a function of the speed with which cells move, as well as the size of the region where movement is present. Thus, the average difference in pixel intensity between neighboring frames can be seen as a surrogate parameter describing the overall motion present within the field of view between two time points.

Accordingly, raw image data were loaded onto ImageJ/Fiji (Rueden et al., 2017; Schindelin et al., 2012) from which the motion trace was obtained and subsequently loaded into IGOR Pro (v8.04, Wavemetrics) where a custom‐built program was used to analyze each trace in a semi‐automatic fashion. After baseline adjustment to zero by means of a linear fit, a threshold of 0.1 was used to identify periods of motion, from which the maximum amplitude (peak amplitude), the width of each motion period (contraction cycle duration), and their frequency were determined and subsequently averaged over the entire trace. Statistics for each sample for a given day were derived from eight traces. Statistical error is given as standard error of the mean (SEM).

### 2.3 Live cell imaging of mitochondria

#### 2.3.1 Sample preparation

Cell monolayers of iCM and mCM were cultured in imaging chambers (µ‐Slide 8 well, #80826; Ibidi, GmbH) for 7 days and subsequently incubated in the presence of mitochondria‐specific fluorescent probes for 30 min at 5% CO_2_. MitoTracker^TM^ Green FM (#M7514; Thermofisher Scientific) served as a membrane‐potential insensitive dye and was used at a concentration of 100 nM (0.01% DMSO [v/v]), while JC‐1 (#T3168; Thermofisher Scientific) served as a membrane‐potential‐dependent dye and was used at 10 µg/ml (0.2% DMSO [v/v]). After incubation, cells were washed with warm basic maintenance medium. MitoTrackerTM‐labeled cells were also labeled with Hoechst 33342 (#R37605; Thermofisher Scientific) for the identification of nuclei.

#### 2.3.2 Data acquisition

Epifluorescence microscopy images were obtained with an Eclipse Ti‐ microscope (Nikon) controlled by NIS‐Elements AR (v5) software (Nikon) equipped with an iXon+897 EMCCD camera (Andor). Temperature (37°C) and atmosphere composition (15% O_2_, 5% CO_2_) and humidity were kept constant during measurements by means of a stage‐top incubator (Okolab). Fluorescence of MitoTracker^TM^ Green FM was captured using a 480/30 nm excitation filter and a 535/45 nm emission filter. Imaging of JC‐1 labeled cells involved imaging cells at two different wavelengths to account for the presence of JC‐1 monomers (480/30 nm excitation and 535/45 nm emission) and JC‐1 aggregates (540/25 nm excitation and 605/55 nm emission).

#### 2.3.3 Data analysis

All images were processed using Fiji/ImageJ (Rueden et al., 2017; Schindelin et al., 2012). Images of cells labeled with MitoTracker^TM^ Green FM were automatically segmented using the minimum cross entropy thresholding algorithm (Li & Lee, 1993) from which the size of detected particles and their mean fluorescence intensities were obtained. The dataset for images labeled with JC‐1 comprised measurements at two wavelengths for the same section of the monolayer, and a ratiometric measurement was made at the pixel level by dividing signal of the JC‐1 aggregate channel by the JC‐1 monomer channel. The mean ratio intensities from each imaged area were then used to compare iCM and mCM monolayers. Statistical analysis of the data was made in Igor Pro (v8.04; Wavemetrics) and significance assessed using the Student's *t*‐test.

### 2.4 Western blot

#### 2.4.1 Sample preparation

Protein extracts were prepared by first dissociating the cells from the basement membrane matrix to which they were attached using dispase II (#D4693; Sigma‐Aldrich) for 10 min at 37°C. The cells were then harvested into a conical tube and resuspended into 10 ml PBS, centrifuged at 500× RCF for 5 min, and the pellet resuspended in RIPA buffer (#89900; ThermoFisher Scientific) supplemented with enzyme inhibitors (Halt^TM^ protease and phosphatase inhibitor cocktail, #78444; ThermoFisher Scientific) and containing 50 mM dithiothreitol. Extracts were then incubated for 5 min at 95°C followed by centrifugation at 21,000× RCF for 5 min to remove any insoluble material and the supernatant was stored at −20°C until used. Western Blot samples were prepared by mixing 13 µl of protein extract aliquots (diluted in PBS if required) in 5 µl sample buffer (Novex NuPAGE LDS‐Sample Buffer [4×], # NP0007; ThermoFisher Scientific) to which was added 2 µl of sample reducing agent (NuPAGE Sample Reducing Agent [10×], # NP0009; ThermoFisher Scientific). Samples were subsequently heated to 70°C for 10 min prior to electrophoresis.

#### 2.4.2 Electrophoresis and data acquisition

Samples were loaded onto 4%–12% polyacrylamide Bis–Tris mini‐gels (NuPAGE, #NP0336BOX; ThermoFisher Scientific), accompanied by a pre‐stained molecular weight marker (PageRuler Plus, #26619; ThermoFisher Scientific). Protein electrophoresis was carried out using a MES‐based running buffer (NuPAGE MES SDS Running Buffer [20×], #NP0002; ThermoFisher Scientific) supplemented with an antioxidizing agent (NuPAGE Antioxidant, #NP0005; ThermoFisher Scientific) to maintain strong reducing conditions throughout electrophoresis. Protein in gels was then transferred to nitrocellulose membranes (Amersham Protran, #GE‐10600004; Sigma‐Aldrich) using a semi‐dry electroblot system (Trans‐Blot SD; Bio‐Rad), followed by staining with 0.5% (m/v) Ponceau S and 1% (v/v) acetic acid for 10 min at room temperature and then partially de‐stained by washing 3× with ultrapure water and then photographed using a gel/blot imaging system (Peqlab Biotechnologie GmbH) to assess protein load. After imaging, the membrane was completely destained with 0.1 N NaOH, followed by rinsing 2× with ultrapure water, after which it was blocked with 5% skimmed milk dissolved in TBS‐T buffer for 1 h at room temperature, followed by washing twice with TBS‐T buffer and then incubated overnight at 4°C with mild agitation in TBS‐T‐buffer with 3% BSA and 0.05% sodium azide and in the presence of the following primary antibodies: anti‐α‐sarcomeric actin (clone 5C5, 1:5000 dilution, #A2172; Sigma‐Aldrich); anti‐α‐smooth muscle actin (clone 1A4, 1:2000 dilution, #MAB1420; R&D Systems); anti‐β‐actin (clone 13E5, 1:1000 dilution, #4970; Cell Signaling); anti‐desmin (clone DE‐U‐10, 1:500 dilution, #ma1036; BosterBio); anti‐cardiac Troponin T (cTnT) (clone 13‐11, 1:1000 dilution, #564766; BD Biosciences); anti‐α/β‐Tubulin (polyclonal, 1:1000 dilution, #2148; Cell Signaling). After incubation with primary antibodies, membranes were washed with TBS‐T 4 × 5 min and further incubated for 1 h in TBS‐T buffer at room temperature in the presence of secondary antibody conjugated with horseradish peroxidase, specifically anti‐mouse IgG (diluted 1:20,000, #115‐035‐062; Dianova) or anti‐rabbit IgG (diluted 1:20,000, #111‐035‐144; Dianova). Membranes were then washed with TBS‐T 3 × 5 min and photographed on gel/blot imaging system (Peqlab Biotechnologie GmbH) using an appropriate chemiluminescence reagent (SuperSignal West Pico PLUS, #34580; ThermoFisher Scientific).

#### 2.4.3 Data analysis

Data analysis was done using Fiji/ImageJ (Rueden et al., 2017; Schindelin et al., 2012), involved three biological replicates per sample, and intensity of detected bands in each lane was corrected for the total protein loaded using the corresponding images of membranes stained with Ponceau S. Statistical significance was assessed using a one‐way ANOVA followed by Tukey's HSD pairwise multi‐comparison test available in the IGOR Pro software package (v8.04; Wavemetrics).

### 2.5 Fluorescence microscopy of cardiomyocyte and cytoskeletal markers

#### 2.5.1 Sample preparation

Cell monolayers cultured in imaging chambers (µ‐Slide 8 well, #80826, Ibidi, GmbH) were fixed and fluorescently labeled using the human cardiomyocyte immunocytochemistry kit (#A25973; ThermoFisher Scientific) and following the manufacturer's instructions. Briefly, after aspiration of medium, cells were fixed for 15 min at room temperature, washed, and then permeabilized for another 15 min at room temperature. After removal of the permeabilization solution, blocking solution was added and cells incubated for 30 min at room temperature, after which they were labeled with primary antibodies overnight at 4°C, washed 3× and incubated with secondary antibody for 1 h at room temperature. When required, cells were further labeled with rhodamine‐phalloidin (#R37112; Thermofisher Scientific) to detect F‐actin and the last washing step was preceded by incubation with Hoechst 33342 (NucBlue^TM^, #R37605; ThermoFisher Scientific) for DNA detection. The primary antibodies used consisted of mouse anti‐ smooth‐muscle‐α‐actin (clone 1A4, #MAB1420; R&D Systems), mouse anti‐cardiac‐α‐actinin (clone EA‐53, #MA1104; Boster Bio), as well as mouse anti‐cardiac‐troponin T and rabbit anti‐nkx2.5 from the human cardiomyocyte immunocytochemistry kit (#A25973; ThermoFisher Scientific). Alexa Fluor^®^ 488 donkey anti‐mouse and Alexa Fluor^®^ 594 donkey anti‐rabbit from the human cardiomyocyte immunocytochemistry kit (#A25973; ThermoFisher Scientific) were used as secondary antibodies. Primary and secondary antibodies were used diluted at 1:500 and 1:250, respectively.

#### 2.5.2 Data acquisition

Epifluorescence microscopy images were obtained with an Eclipse Ti‐ microscope (Nikon) controlled by NIS‐Elements AR (v3.22) software (Nikon) equipped with an iXon+897 EMCCD camera (Andor). For each channel, all camera and microscope settings were kept constant across all samples.

#### 2.5.3 Data analysis

All images were processed using Fiji/ImageJ (Rueden et al., 2017; Schindelin et al., 2012) which involved an initial step of converting them to an 8‐bit color scale, followed by despeckling to remove spurious noise.

To assess changes in the expression of the nuclear protein Nkx2.5, images of cells labeled with the DNA‐binding fluorophore Hoechst 33342 showing nuclei were used to segment images labeled for Nkx2.5 from which the average fluorescence intensity of each nucleus was extracted. The fluorescence intensity reported is the average of the fluorescence intensity of all instances of nuclei in the Nkx2.5 channel.

To quantify differences in the levels of cytoskeletal proteins, images of cells labeled with rhodamine‐phalloidin were used to create a mask using Huang's fuzzy thresholding method. In the absence of rhodamine‐phalloidin staining, fluorescence from immunolabeled cTnT was used to create the mask. This procedure allowed for the segmentation of cellular patches, each with a different area $A_{i}$ and average fluorescence intensity ${\bar{F}}_{i}$. Combining every *i* instance of cell‐patches within an image, the corresponding weighted average fluorescence intensity (*F*) here reported follows the expression:

$$F=\frac{\sum \left(A_{i}\times \bar{F_{i}}\right)}{\sum \left(A_{i}\right)}$$

Statistical analysis was performed in Igor Pro (v8.0.4.2; Wavemetrics) using a one‐way ANOVA to capture global *p*‐values, followed by multiple pairwise comparisons with Tukey's HSD test.

### 2.6 Atomic force microscopy

#### 2.6.1 Setup and sample preparation

To determine the Young's modulus of cells in the adherent state, we performed indentation experiments using an atomic force microscope (Nanowizard 3; JPK Instruments) and tipless cantilevers (HQ:CSC38; MikroMash) with a nominal spring constant of 0.03 N/m that were experimentally calibrated using the thermal method (Hutter & Bechhoefer, 1993). Cantilevers were modified using a UV‐curing adhesive (#NOA63; Norland Products) to attach a single SiO_2_ spherical probe (MicroParticles GmbH) with a diameter of 4.77 ± 0.2 µm (mean ± SD) to the very tip of the lever.

Cells were cultured in glass‐bottom WillCo‐dishes (#GWST‐5040; Willco wells) previously coated with Geltrex^TM^ (see above) and cultured for 2–3 days (CPC) or 7–8 days (iCM and mCM) prior to measurements. On the day of measurement, cells were washed with basic maintenance medium and incubated in the same medium for 30 min (37°C/5% CO_2_) in the presence of 1 µM cytochalasin D (CytoD) and 0.245% (v/v) dimethyl sulfoxide (DMSO), or only with 0.245% (v/v) DMSO (vehicle control) or in the absence of either of these compounds (negative control). Following the incubation period, cells were again washed with basic maintenance medium and immediately probed.

#### 2.6.2 Data acquisition

Measurements of cell elastic properties for each cell type at a given treatment condition were done in duplicate. Cell monolayers were probed by pressing on the cells with a cantilever modified with a spherical indenter at a speed of 2 µm/s and with a force setpoint of 1 nN in a series of 3×3 matrices each covering an area of 10×10 µm^2^. All measurements were done in maintenance medium at room temperature for up to 1 h.

#### 2.6.3 Data analysis

The complete dataset comprised thousands of force‐indentation curves that were individually modeled after Hertz elastic model for a spherical indenter (Lin et al., 2007):

$$F=\frac{4{\text{ER}}^{1/2}}{3\left(1‐\nu^{2}\right)}\delta^{3/2}$$

where the measured indentation force (*F*) is proportional to the indentation depth (*δ*) and depends on the material's Young's modulus of elasticity (*E*), it's Poisson's ratio (*ν*, here set to 0.5), and the radius of the indenter (*R*). Given that some indentation events can experimentally deviate from some model assumptions, force‐indentation curves were selected for analysis given two criteria: a root mean square of residuals smaller than 25 pN, and contact point deviation smaller than 100 nm (see Table S1). From these, 1.5% of the curves corresponded to values of *E* outside the range between 1 × 10^2^ and 1 × 10^4^ Pa and were excluded resulting in a dataset comprised of 2233 measurements. Statistical significance between groups was assessed through a linear mixed‐effect model using the “lme4” (Bates et al., 2015) package in R (v. 4.1.0). The model takes variance of the Young's modulus to result from the linear combination of fixed effects related with cell type, treatment, and interaction between them, with random affects attributed to measurement day. Assessment of normality and homoscedasticity of residuals was ensured by visually inspecting QQ‐plots and residuals versus fitted plots and global *p*‐values were obtained using ANOVA‐like type 3 sum of squares. We report least‐squared means and SEM obtained through the “emmeans” package (Lenth, 2020).

### 2.7 Real‐time deformability cytometry

#### 2.7.1 Sample preparation

Cells cultured in 12‐well tissue culture plates coated with GelTrex to a density of 0.1 × 10^6^ cells/cm^2^ were enzymatically detached into a single cell suspension by incubating them with 190 μl of TrypLE (#A1217702; Thermo Fischer Scientific) for 5 min at 37°C, after which the liquid suspension was discarded, and the procedure repeated for another 5 min. The reaction was stopped with the addition of 2 ml of RPMI 1640 medium without glutamine (Biowest) containing 10% fetal calf serum. The supernatant was subsequently aspirated and centrifuged at 200× RCF for 5 min, and the resulting cell pellet was re‐suspended in 100 µl of MC‐PBS buffer consisting of PBS (without Ca^2+^ and Mg^2+^) containing 0.6% (w/v) methyl cellulose (MC; Sigma Aldrich). The typical cell density in each sample was approximately 1 × 10^6^ cells/ml.

#### 2.7.2 Data acquisition

Using the RT‐DC AcCellerator system (Zellmechanik Dresden) as described earlier (Mietke et al., 2015; Otto et al., 2015) we determined the Young's modulus of the different cell types using single cell deformation and size as read‐out parameters. Briefly, samples containing suspended cells were injected into a polydimethylsiloxane (Sylgard 184, VWR) microfluidic chip consisting of a narrow 300 µm‐long channel with a 30 µm × 30 µm square cross‐section where cells undergo deformation due to hydrodynamic shear and normal stresses. By means of a high‐speed CMOS camera (MC1362; Mikrotron), single cells are imaged inside and outside the channel to record their deformed and undeformed states, respectively.

#### 2.7.3 Data analysis

On‐the‐fly image analysis is performed using the ShapeIn control software (v2.0.1; Zellmechanik Dresden) to determine cell deformation and size. These parameters were then used by ShapeOut analysis software (v0.8.4; Zellmechanik Dresden) that assumes steady‐state conditions to calculate the Young's modulus of each cell by coupling the hydrodynamic stress distribution around the cell to linear elasticity theory (Mietke et al., 2015). Data from three biological replicates per cell type and comprising thousands of single cell measurements were compiled into a single data frame and statistically processed in RStudio (v. 1.2.5033). The “lme4” package was used to build a two‐level linear mixed‐effect model where the cell type was taken as a fixed effect and sampling contributed to random effects (Bates et al., 2015). Statistics are reported with respect to least square means and standard error, which together with *p*‐value were calculated through the “emmeans” package (Lenth, 2020). Unless otherwise stated, all reported values correspond to the mean and the given error refers to the SEM.

## 3 RESULTS

### 3.1 iCM cardiomyocytes display a more immature contraction phenotype

**FIGURE 1:**
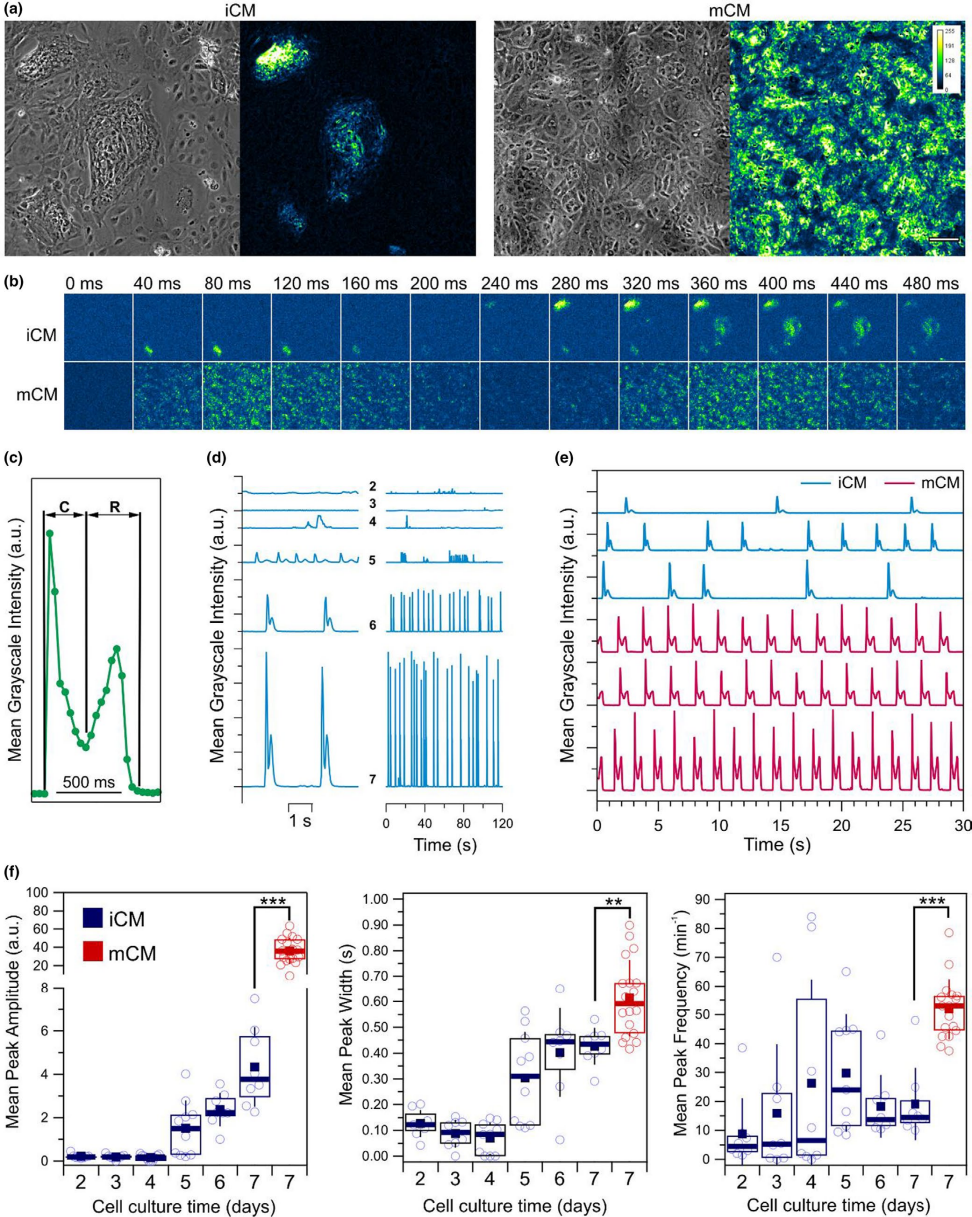
Motion analysis of iCM and mCM cardiomyocyte monolayers. (a) Typical phase‐contrast images (left) and time‐integrated motion signals over a 1 min period (right) for iCM and mCM monolayers. Cells in iCM cultures tend to form tight cell clusters that often exhibited contractile motion, while in mCM motion activity was typically more homogeneously distributed. Color scale denoting signal intensity, and scale bar of 100 µm are applicable to all respective subpanels. (b) Output of the motion detection over 13 consecutive frames spanning a period of 480 ms for the examples shown in (a) reveals differences in motion synchronicity within iCM and mCM monolayers. Motion in mCM monolayers clearly display two moments related to a contraction and a relaxation phases, which in iCM were typically more well defined at the level of the individual cell clusters rather than between clusters. (c) A typical spatially averaged contractile motion signal displays two moments related with the contraction (C) and relaxation (R) phases. Each point corresponds to the mean motion signal intensity across the imaged field of view. (d) Evolution of motion signals in iCM monolayers across different days of culture indicate an increase in contraction frequency and increased levels of motion across the monolayer. (e) Comparison of motion traces between iCM and mCM on the seventh day of culture. (f) Evolution of the mean motion peak amplitude, width, and frequency as a function of cell culture period for iCM and mCM. In plots, open circles represent mean values obtained from a single motion trace over a period of 120 s (*n* = 8), boxes show the interquartile range, error bars show standard deviation, filled squares and thick horizontal lines indicate means and medians, respectively. Statistical significance assessed by the Student's *t* test (**p < 0.01; ***p < 0.001)

To assess the differences between iCM and mCM cardiomyocytes, we began by looking at their spontaneous contraction properties using real‐time phase‐contrast video microscopy. We followed the development of the dynamic behavior of iCM during the 7‐day culture period, at the end of which we compared with mCM cultured for the same time (Figure 1). Over this 7‐day period, iCM cells formed clusters of densely packed cells that were sparsely dispersed, while mCM cells were more homogeneously distributed (Figure 1a). Motion analysis was performed using in‐house developed algorithm which we used earlier to describe cardiomyocyte contractility following cytoskeletal remodeling (Pires et al., 2019). Given that the algorithm is not based on edge detection, but instead in light intensity fluctuations, it is highly sensitive to movements that are below the resolution limit of the imaging system, allowing for large areas of a monolayer to be interrogated. By computing the mean difference in grayscale intensity between consecutive frames we obtain a surrogate parameter that assesses the extent of motion in the field of view over time (Figure S1). Absence of motion between neighboring frames results in motion values that are at noise levels. We can then integrate the result from the motion‐detection over the measurement period (120 s), allowing us to map the regions within a monolayer that are subject to motion (Figure 1a). We observed that while in iCM only in discrete regions of the monolayer we could detect movement (Figure 1a, left); motion in mCM cultures were typically more uniformly distributed (Figure 1a, right). Whenever more than one active cluster of iCM cells could be imaged, we often detected lack of synchronicity between them (Figure 1b; Video S1). By monitoring the location of motion signals over time we could detect delays of hundreds of milliseconds that were sometimes coupled with apparent phase differences. For example, Figure 1b shows motion signals emerging in a localized cell cluster in the lower‐left quadrant, with a second cell cluster in the upper‐left quadrant initiating motion 200 ms after. By contrast, motion in mCM was more synchronous, with the first motion signals emerging throughout the whole field of view (Video S2). Characteristic of the mCM monolayers, and indeed of each individual iCM cell cluster, was a motion profile that had two well‐defined moments corresponding to a contraction phase, and a relaxation phase (Figure 1b,c). Taking the mean motion signal intensity in each frame, we summarize the detected motion in the form of a single surrogate parameter whose evolution over a contraction‐relaxation cycle acquires the shape of a peak‐doublet (Figure 1c). This allowed us to easily evaluate the emergence of contractile motion during differentiation, which we typically detected on the 5th day of culture, with signals increasing in amplitude over the remaining culture days (Figure 1d). However, after 7 days of culture, the motion traces of iCM and mCM show clear differences with respect to amplitude, frequency, and regularity (Figure 1e). Seven days after seeding cells, the amplitude of mCM was significantly higher than that of iCM (Figure 1e,f). Similarly, the mean width of the peak doublets (mean contraction cycle duration) in iCM increased reaching 0.42 ± 0.07 s on day 7, which is significantly lower than at 0.61 ± 0.14 s recorded for mCM after the same culturing period (Figure 1f). Finally, with respect to the contraction frequency of cardiomyocytes, a similar trend was observed. The mean number of motion peaks per min increased in iCM with the culturing period reaching 19 ± 12 peaks/min on the 7th day of culture, which significantly contrasts with 52 ± 12 peaks/min registered for mCM (Figure 1e,f).

### 3.2 iCM cardiomyocytes show reduced mitochondrial activity

Changes in contractility can be associated with differences in the intracellular energy pool for which mitochondria are the major contributor. Given that cardiomyocyte differentiation invokes metabolic adjustments that reflect themselves in cellular ATP production levels, we sought to clarify if the differences in contractility could be correlated to changes in the organization and activity of mitochondrial networks (MNs) in iCM and mCM. Doing so we used two distinct probes: the MitoTracker^TM^ Green FM and JC‐1 probes, both of which accumulate in active mitochondria but only the latter is dependent on the mitochondria inner membrane potential (Mathur et al., 2000). Regarding JC‐1, once inside mitochondria, the high concentrations of the probe, the pH, and the ionic strength of the environment contribute to the formation of so called “J‐aggregates” which have distinct optical properties compared with monomeric form of the compound. Thus, the ratio of the JC‐1 aggregates over JC‐1 monomers is a useful measure of the proton electrochemical gradient across the inner mitochondrial membrane. By contrast, MitoTracker Green FM signals are better suited to study the localization and morphological properties of mitochondria given the fluorescence signal is unaffected by details of mitochondrial activity.

**FIGURE 2:**
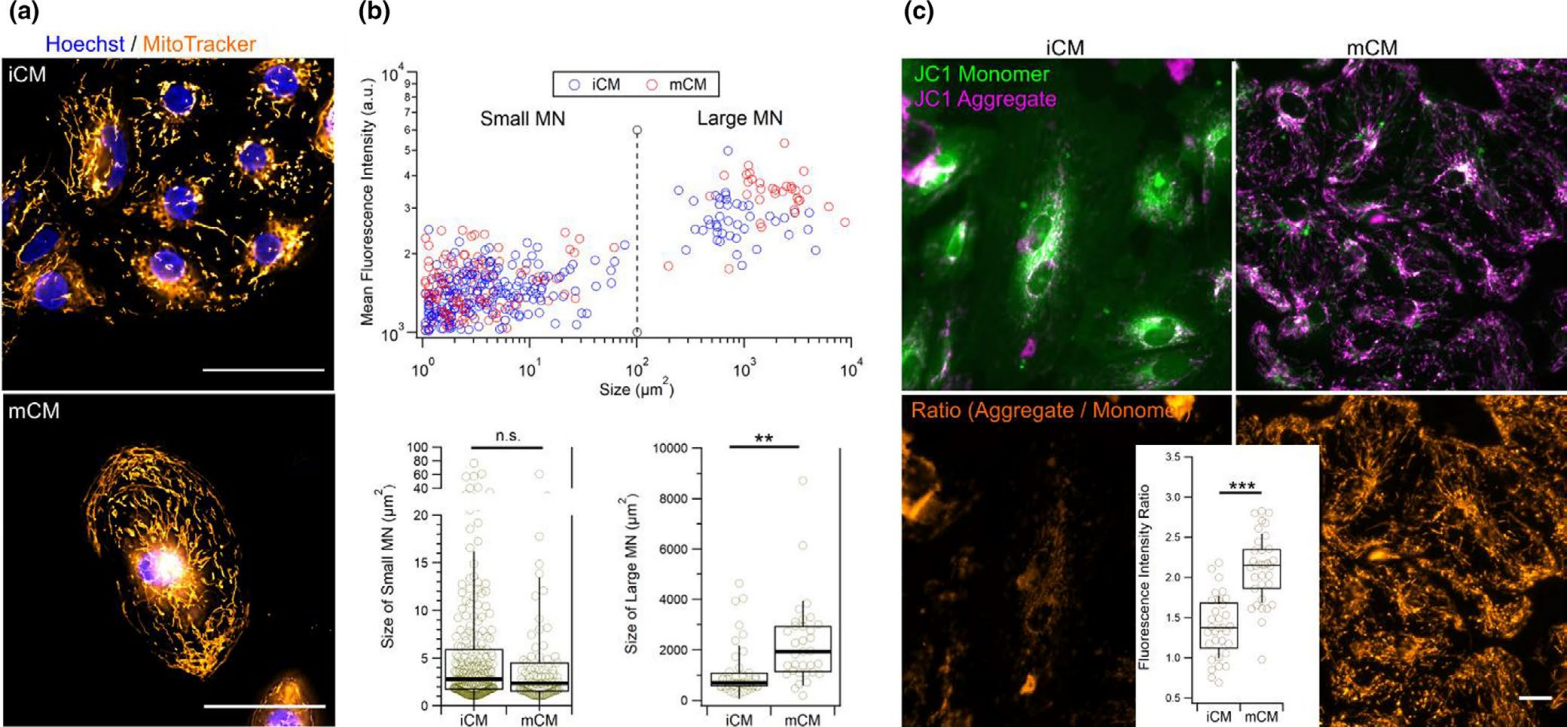
Live cell fluorescence imaging of mitochondria. (a) Live cell imaging with the membrane potential independent mitochondrial probe MitoTracker^TM^ Green FM shows topographical and morphological differences in mitochondrial networks. Mitochondria in iCM typically accumulate in the perinuclear region while in mCM they also span toward the cellular cortex. Scale bar is 50 µm and applies two both subpanels. (b) Direct correlation between the size of mitochondrial networks (MN) and their mean fluorescence intensity when imaged with MitoTracker^TM^ Green FM allows classification of MN into small and large MN with sizes of up to 100 µm^2^, and above 100 µm^2^, respectively (top). Comparing the sizes of mitochondrial networks (MN) between iCM and mCM shows that large MNs in mCM are significantly larger, but no significant size differences were found for small MNs (bottom). (c) Live cell imaging of cells labeled with JC‐1 mitochondrial membrane potential‐dependent fluorescence probe reveals clear differences between iCM and mCM in signals from JC‐1 monomers and JC‐1 aggregates shown in composite images (top). Analysis of ratio image intensities (bottom) indicates significantly higher activities in mCM mitochondria. Scale bar is 20 µm and applies to all subpanels. Statistical significance assessed by the Student's *t* test (**p < 0.01; ***p < 0.001; n.s., non‐significant)

MitoTracker Green FM allowed us to visualize the distribution of mitochondria in iCM and mCM (Figure 2a,b). We observed accumulation of mitochondria in the perinuclear region in both cell types, but that a dense network of mitochondria also appeared in the cortical region of mCM (Figure 2a). By determining the size of the MN, and setting the threshold at 100 µm^2^, we could classify them into small and large MN (Figure 2b, top). Doing so we could see no difference in the size of small MN from iCM and mCM, but a significant increase in the size of large MN was detected for mCM (Figure 4b, bottom). Interestingly, we also observed an increase in the fluorescence signal for MN from mCM both small and large, which we attribute to a likely increased mitochrondrial density (Figure S2). Inspecting cells with the JC‐1 probe revealed further differences between the mitochondria of both cell types, with higher conversions of JC‐1 monomer to JC‐1 aggregates apparent for mCM cells compared with iCM (Figure 2c). Thus, our findings indicate that compared with iCM, the mCM networks of mitochondria are larger, and denser and are more active, with a higher inner membrane potential (Figure S2).

### 3.3 CPC, iCM and mCM predominantly express different actin isoforms

**FIGURE 3:**
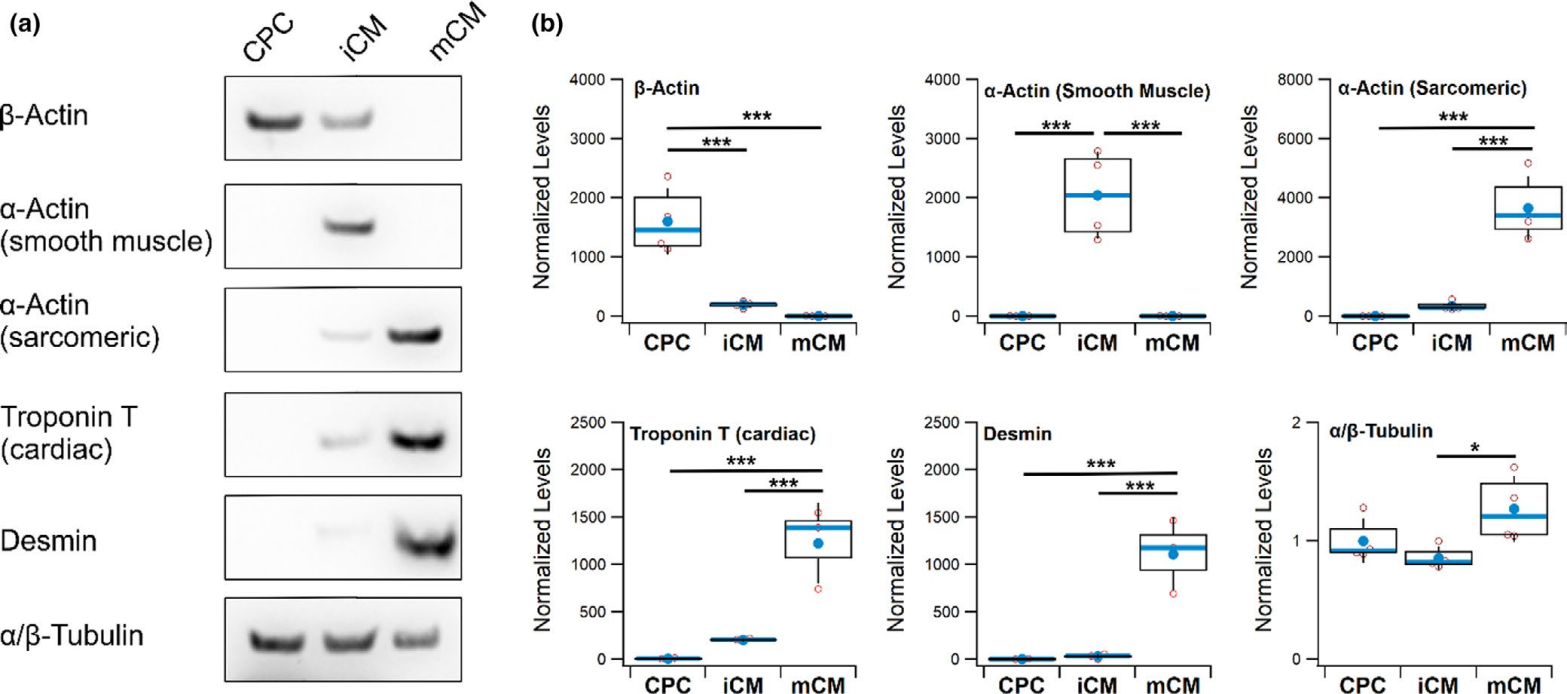
Western Blot detection of cytoskeletal proteins. (a) Typical Western Blots obtained for six different cytoskeletal proteins in samples of cardiac progenitor cells (CPC), immature (iCM) and more differentiated (mCM) cardiomyocytes. (b) Evaluation of the differences in expression across three to four biological replicates. Variations in protein load were corrected using total protein derived from Ponceau S staining of the blotted membranes and final results are presented normalized to the mean expression of the CPC samples, except for β‐actin where the mean of the mCM sample was used. In boxplots, the box corresponds to the interquartile range, error bars show standard deviation, with mean and median represented by a filled circle and centerline, respectively. Statistical analysis performed with ANOVA followed by Tukey's HSD pairwise multi‐comparison test (*p < 0.05; ***p < 0.001)

Having established that iCM and mCM display significant differences in their contractile behavior, we sought to find possible links to their cytoskeletal composition using Western Blot (Figure 3a). Indeed, we found that mCM displayed higher levels of sarcomere‐associated proteins such as cTnT, desmin, and sarcomeric α‐actin, which were undetected in CPC, with iCM having displayed intermediate levels of expression (Figure 3b). These differences that correlate with differences in motion properties between the cells appeared to reflect also on the specific actin isoform being expressed, with CPC having the highest levels of β‐actin, while iCM and mCM showed the highest levels of smooth muscle α‐actin, and sarcomeric α‐actin, respectively (Figure 3a). In addition, we found that while iCM revealed uniquely significant levels of smooth muscle α‐actin, it was possible to detect all three isoforms of actin in these cells (Figure 3a).

**FIGURE 4:**
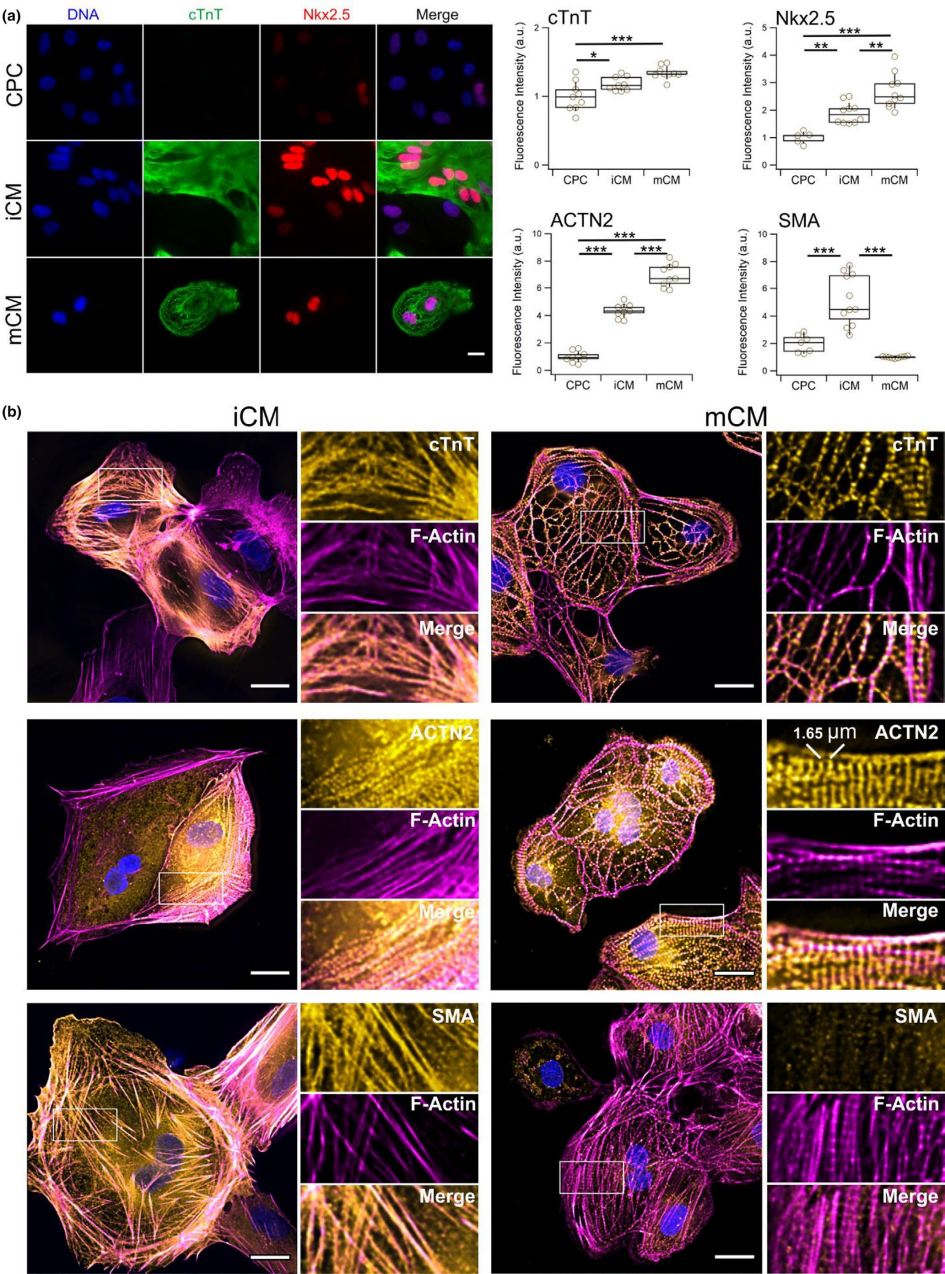
Detection of cardiomyocyte and cytoskeletal markers by fluorescence microscopy. (a) Fluorescence microscopy images of cardiac troponin T (cTnT) and Nkx2.5 for samples of cardiac progenitor cells (CPC), and immature (iCM) and more differentiated (mCM) cardiomyocytes (left) and image‐based quantification of cTnT, Nkx2.5, sarcomeric α‐actinin (ACTN2), and smooth muscle α‐actin (SMA) (right). Results suggest that iCM are an intermediary state of differentiation between CPC and mCM uniquely displaying high levels of SMA. Scale bar is 20 µm applicable to all subpanels. In boxplots, *n* = 9 for each sample, boxes indicate interquartile range, error bars show standard deviation. (b) Composite fluorescence microscopy images of iCM and mCM cardiomyocytes labeled for F‐Actin with rhodamine‐phalloidin and immunolabeled for cardiac troponin T (cTnT), sarcomeric α‐actinin (ACTN2), smooth muscle α‐actin (SMA). Insets show a magnified view of single channel information from the highlighted rectangular areas. All markers display high levels of integration within the F‐actin cytoskeletal network, however, recognizable patterns typical of more differentiated cardiomyocytes are typically apparent in mCM. Scale bars are 20 µm. Statistical analysis performed with ANOVA followed by Tukey's HSD pairwise multi‐comparison test (*p < 0.05; **p < 0.01; ***p < 0.001)

iCM display early signatures of sarcomere assembly. Following the observation of differences in contractile activity between iCM and mCM, as well as in their displayed levels of sarcomeric proteins that appeared associated to a switch in actin isoform expression, we wondered how this was reflected in the sarcomere structure. Resorting to fluorescence microscopy, we began by confirming western‐blot data with images evidencing protein levels that correlated with the extent of cardiomyocyte differentiation. While levels of cTnT were higher in cardiomyocytes compared with CPC, but no significant differences could be found between iCM and mCM. However, levels of Nkx2.5, a critical transcription regulator tightly associated with the expression of several cardiomyocyte‐specific genes (Anderson et al., 2018), and sarcomeric α‐actinin (ACTN2) showed significant increase that correlates with the extent of cardiomyocyte differentiation (Figure 4a). Importantly, however, the levels of these proteins did not appear to be homogeneously distributed in iCM as they were in mCM (Figure S3).

Next, we looked at the incorporation of cTnT in the actin cytoskeleton using phalloidin to label actin filaments. Images of cTnT‐labeled mCM displayed a banded pattern that followed much of the contour of actin filaments and is indicative of assembled sarcomeres (Figure 4b, top right). Unlike the case of mCM, we could not observe a similar banding structure along the actin filaments of iCM. Instead, we observed a much more subtle pattern with detection of cTnT along actin‐filaments that suggest an early stage of assembly of sarcomeres. (Figure 4b, top left). A similar observation could be made when cells were labeled for sarcomeric α‐actinin (ACTN2) which in the case of mCM showed a defined band structure (Figure 4b, middle right) that was absent in iCM, but where, like for cTnT, signs of recruitment of the protein to the filaments were detected (Figure 4b, middle left). The sarcomeric α‐actinin is an integral component of Z‐disks which define the unit length of a sarcomere. By analyzing the distance between bands in images of mCM cells, we determined a length of 1.65 ± 0.03 µm (mean ± SEM), which is smaller than that of typical primary adult cells, but in line with previous reports on iPSC‐derived cardiomyocytes (Figure S4; Lemcke et al., 2020). Finally, we wanted to ascertain if the expression of smooth muscle α‐actin (SMA) detected in Western Blots was a feature of the iCM cytoskeleton (Figure 4b, bottom). Images confirmed Western Blot data in indicating that iCM represents a particular state of differentiation characterized by high levels of SMA (Figure 4a, right). The signal from SMA appeared to co‐localize with large sections of the F‐actin network, suggesting it to be a major structural cytoskeletal component of iCM, but not so in mCM (Figure 4b), nor in CPC (Figure S4).

### 3.4 Cell stiffening is associated with differentiation of cells with cardiomyocyte phenotype

To further scrutinize the process of cardiomyocyte differentiation and maturation, we characterized the cells with respect to their elastic properties by measuring its Young's modulus using two fundamentally different techniques: atomic force microscopy (AFM) and RT‐DC. While AFM is typically more suited for adherent cells and provides highly localized measures of elasticity, RT‐DC is a microfluidic technique that measures cells in suspension and provides mechanical information averaged over the entire cell volume. Despite these differences, we found earlier the two techniques providing good agreement with each other when measuring the Young's modulus of iPSC‐cardiomyocytes under conditions involving actin remodeling (Pires et al., 2019).

**FIGURE 5:**
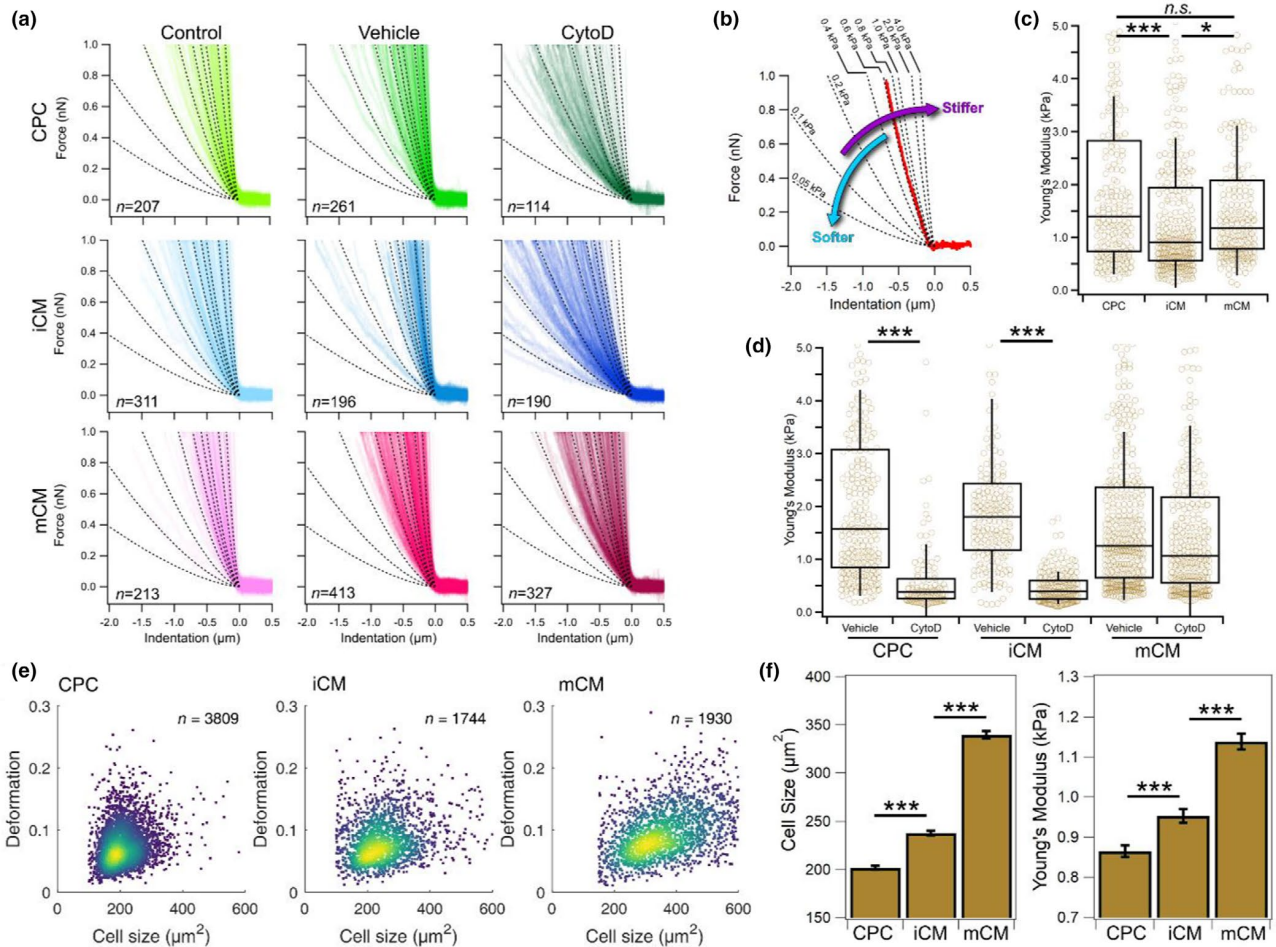
Analysis of cell mechanical properties by AFM and RT‐DC. (a) Superposition of multiple force‐indentation curves from AFM‐based colloidal force spectroscopy obtained from two biological replicates of cardiac progenitor cells (CPC), immature (iCM) and more differentiated (mCM) cardiomyocytes across different sample conditions of negative control (control), DMSO vehicle control (vehicle), and 1 µM cytochalasin D (CytoD). Dotted lines represent isoelasticity lines and serve as reference to compare data between plots. (b) Example of a single force indentation curve (shown in red) plotted together with isoelasticity lines (dotted) annotated for their corresponding Young's modulus. The inclination of the curve is proportional to the stiffness of the probed area. (c) Comparison of AFM measurements for negative control samples of CPC, iCM and mCM showing significant differences in Young's modulus between the iCM and either CPC or mCM, but not between CPC and mCM. (d) AFM measurements to probe the effect of 1 µM CytoD (CytoD) compared with vehicle control (vehicle) shows a significant decrease in the Young's modulus for CPC and iCM, but not for mCM. (e) Typical RT‐DC scatter plots of deformation as a function of cell size for the CPC (left), iCM (centre) and mCM (right) show that while changes in deformation appear subtle, there are clear differences in the size of the different cell types. (f) Evaluation of RT‐DC data from three biological replicates show a significant increase in cell size and Young's modulus that positively correlates with the extent of cardiomyocyte differentiation. The total number of single measurements were: 10,761 for CPC, 4451 for iCM, and 5087 for mCM. In boxplots, boxes indicate interquartile range, error bars show standard deviation. Bar charts show estimated marginal means and error bars represent standard error of means. Statistical analysis was performed using linear mixed‐effect models (*p < 0.05; ***p < 0.001; n.s., non‐significant)

Using an AFM setup, we mechanically interrogated CPC, iCM and mCM by pressing cells with a micron‐size spherical probe attached to a flexible force‐calibrated cantilever (Figure S4). Given the changes in actin isoform composition across the three cell types, we wondered how CytoD might differentially impact the mechanical properties of the cells (Figure 5a). The result of each single measurement is a force‐indentation curve where upon contact the probe is pressed hundreds of nanometers into the cell resulting in an exponential rise in force that is dependent on the stiffness of the region being probed (Figure 5b). Accumulating hundreds of measurements for each condition, we begun looking at the elasticity of cells in the absence of any additional exogenous compound (negative control). Analysis of CPC revealed a Young's modulus *E* of 1.41 ± 0.11 kPa, which was not significantly different from the 1.26 ± 0.10 kPa determined for mCM, with only the *E* of iCM showing a statistically significant decrease to 1.03 ± 0.08 kPa (Figure 5c). Interestingly, addition of DMSO as a vehicle control for the CytoD treatment resulted in a significant increase in *E* of iCM to values closer to those of CPC (Table S1). The incubation with 1 µM CytoD resulted in a significant decrease in the Young's modulus of CPC and iCM, but not of mCM (Figure 5d).

Inspecting conventional RT‐DC scatter plots for CPC, iCM and mCM (Figure 5e) suggests that all three cell cultures resulted in homogeneous samples, with no subpopulations being immediately identifiable. Despite this, it is apparent that across samples there are differences in both deformation and size, with changes in the latter being more apparent (Figure 5e; Figure S6). Looking across three biological replicates for each sample, CPC cultured for 2 days in the presence of bFGF to induce their proliferation resulted in cells with a size of 202 ± 2 µm^2^ but became enlarged with differentiation, reaching on average 238 ± 3 µm^2^ for iCM (Figure 5f, left panel). Differences in elasticity were also noticeable with CPC having a Young's modulus of 0.87 ± 0.02 kPa which increased to 0.95 ± 0.02 kPa upon differentiation to iCM (Figure 5f, right panel). A substantial increase in cell size, deformation, and Young's modulus was particularly apparent when iCM and mCM were compared. Note that, except for the first 2 days of culture when CPCs were stimulated to differentiate into cardiomyocytes, the growth conditions were identical between the two cell types for the remaining 5 days of culture, notably the medium and the substrate were the same in both cases. Nonetheless, we observe marked differences between the more immature and more differentiated forms of cardiomyocytes, with the latter being significantly larger with a size of 340 ± 4 µm^2^ as well as significantly stiffer as evidenced by their larger Young's modulus of 1.14 ± 0.02 kPa (Figure 5f).

## 4 DISCUSSION

Understanding the details of cardiomyocyte differentiation and maturation is extremely important for the development of new therapeutic and diagnostic tools (Karbassi et al., 2020). To maximize the physiological relevance of such studies, it is important to be able to integrate data collected at different length scales, ranging from the molecular level, crossing the dimensions of individual cells, and reaching the scale of tissues and organs (Sherman & Grosberg, 2019). Here we aimed at implementing such approach by combining protein data generated by fluorescence microscopy and Western Blot with the characterization of mechanical properties of single cells, while also assessing the collective dynamic behavior of the corresponding cell monolayers. Using iPSC‐derived cell model systems, we focused on the stage of cell differentiation involving the transition of CPC to early‐stage cardiomyocytes (iCM), which we then compared with more differentiated cells (mCM).

Differentiation of CPC to iCM resulted in cells that displayed contractile behavior but details of their motion, such as lack of synchronicity and contraction limited to small clusters, denotes a cell population that is still in the early stages of differentiation. Analysis of mCM allows us to establish that while they too display hallmarks of cardiomyocyte immaturity (such as lack of sarcomere alignment and registration, and short sarcomere lengths) they appear to be, by comparison with iCM, in a more developed state of differentiation. Indeed, as expected for mCM, MNs appeared more developed, not so localized to the perinuclear region and typically showed higher levels of activity compared with iCM (Scuderi & Butcher, 2017). Furthermore, while iCM and mCM did appear to express many of the same cardiomyocyte markers, the levels of such proteins were typically higher in mCM. These differences in protein levels were accompanied by differences in how key sarcomeric proteins were inserted in the actin cytoskeleton. Unlike mCM for which we detected by fluorescence microscopy, a banding pattern in cTnT and ACTN2 labeled cells that is characteristic of developed cardiomyocytes, these patterns were absent in iCM. Nonetheless, our images show recruitment of these proteins to actin filaments, suggesting that indeed iCM are an early‐stage cell type along the cardiomyocyte differentiation pathway. Unique to iCM among the three cell types evaluated in this work was the high levels of smooth muscle actin (SMA), which we observed by both Western Blot and fluorescence microscopy. The latter indicates high levels of incorporation of SMA in the F‐actin network that was only poorly detected in CPC and especially low in mCM. This high amount of SMA appears to be a hallmark feature of very early stage differentiating cardiomyocytes. It has been shown earlier, that during cardiogenesis, the heart transiently expresses smooth‐muscle α‐actin (Hayward & Schwartz, 1986). Another earlier report has also showed that during the embryogenic development of cardiomyocytes, smooth‐muscle α‐actin is temporarily expressed in significant levels before the expression of both cardiac and skeletal α‐actin eventually becomes more predominant (Clément et al., 2007). Thus, the presence of smooth‐muscle α‐actin in iCM, clearly sets this cell type apart from the other two, while marking the onset of a cardiomyocyte phenotype as observed by the levels of other cardiomyocyte‐specific proteins. According to RT‐DC image data, the increased level of differentiation of CPC >iCM>mCM was paralleled by an increase in cell size. Indeed, enlargement of the cardiac tissue is in part driven by hypertrophic growth of cardiomyocytes that acquire unusually large sizes, reaching over 2000 µm^2^ in primary cells (Lai et al., 2011). The sizes we observed are considerably smaller, but the hypertrophic trend is clear.

The cytoskeletal remodeling that we describe here has the potential to impact the mechanical properties of the cells. On this topic, we assayed all three cell types using two very distinct methods: (a) AFM‐based colloidal force spectroscopy, where cells probed are adhered to the surface, and (b) RT‐DC where cells are detached from the surface and measured in suspension. AFM and RT‐DC results are in good agreement with each other in measurements for iCM and mCM (Figure S7). Not only do both methods show the same trend between the two cell types, but the difference between the two methods was only 0.08 and 0.12 kPa for iCM and mCM, respectively. For comparison, earlier reports on the Young's modulus of hiPSC cardiomyocytes by RT‐DC indicate an *E* of 1.25 kPa (a 0.11 kPa difference) (Pires et al., 2019). Here, both techniques show that the more differentiated mCM are significantly stiffer than the more immature iCM cells. In the context of cardiac development, increase in tissue stiffening was reported earlier when the epicardial surface of the left ventricle of mice embryos was compared with those of neonatal ones (Jacot et al., 2010). However, such increase could also be explained by an increased deposition of collagen and other extracellular matrix component which have been shown to contribute more to myocardial stiffness than its cellular content (Perea‐Gil et al., 2015). Here, by looking at the mechanics of single cells in suspension, we clearly demonstrate that cell stiffening is an ongoing process for which we show evidence of being linked to the cytoskeletal changes that accompany differentiation, and not exclusively to their surrounding polymer matrix.

Contrary to iCM and mCM where RT‐DC and AFM data are in good agreement, measurements for CPC show a larger disparity between the two methods (0.54 kPa difference) where the cells in the adhered state appeared to be stiffer. Our data indicate that measuring cells in suspension by RT‐DC renders them softer compared with when they are in the adherent state as measured by AFM. Studies have shown that cells have different susceptibilities concerning their adhesion state that translates to changes in their elasticity (Mierke, 2019). While transition from adherent to suspension does not necessarily involve an increase in cell compliance (Haghparast et al., 2015) an earlier study using an optical stretcher highlighted the role of non‐muscle myosin II (NMII) as a key element in softening cells as they transition from the adherent to the suspension state (Chan et al., 2015). NMII is a motor protein involved in the binding (Hu et al., 2017) and pre‐tensioning of β‐actin filaments (Vicente‐Manzanares et al., 2009). Conceivably, the apparent downregulation of β‐actin in iCM and mCM in favor of higher levels of either the smooth muscle or sarcomeric alpha isoforms results in the emergence of an actomyosin network with different mechano‐regulatory properties. In an earlier AFM study involving β‐actin knock‐out (−/−) and heterozygous (+/−) cells showed that not only do they displayed compensatory behavior involving the upregulation of SMA, but that cells became softer with decreasing levels of β‐actin, and increasing levels of SMA (Xie et al., 2018). Likewise, our AFM data also show that transition from CPC to iCM involving apparent lower‐levels of β‐actin in exchange for higher levels of SMA results in softer cells. Upon detachment, however, the trend reverses and iCM appears stiffer than CPC largely due to mechanical changes in the latter. Thus, it appears that the transition from CPC to iCM involving a softening of cells can be associated with switching of the actin‐isoform expression that results in a cytoskeleton whose structural stability appears more independent in the adhesion state. This last aspect appears to be a shared property between iCM and mCM, whose elastic properties of the cells attached to the surface remain very similar when placed in suspension. However, as shown by fluorescent microscopy, the differences in the cytoskeletal organization of the two cells are rather large. As a consequence, when cells were challenged with 1 µM CytoD, both CPC and iCM show a significant reduction in their Young's modulus. By contrast, the CytoD treatment had no significant impact in the Young's modulus of mCM. CytoD does not actively depolymerizes actin filaments, and instead inhibits filament polymerization, making dynamic filaments that undergo frequent turnover to be more susceptible to its effects. Despite the switching in actin isoform expression from CPC to iCM, and the phenotypic overlap between iCM and mCM, the effects of CytoD in iCM are similar to those in CPC.

In conclusion, we have shown that even at early stages in the process of cardiomyocyte differentiation there is a detectable evolution of contractile activity of cell monolayers that is paralleled by changes in cytoskeletal composition that are coupled to changes in cell elasticity. We provide evidence that these changes can be linked to an altered composition of the actin‐network with the switch of isoform expression from β‐actin to smooth muscle α‐actin associated with the emergence of a cardiomyocyte‐like phenotype and a change in the mechanical properties of the cells that become less sensitive to cell detachment.

## CONFLICT OF INTEREST

OO is co‐founder and shareholder of ZELLMECHANIK DRESDEN distributing real‐time deformability cytometry technology. All other authors do not report any conflict of interests.

## AUTHOR CONTRIBUTIONS

Ricardo H. Pires performed cell culture, microscopy, and contractility experiments. Tung H. Dau performed Western Blots. Nithya Shree performed RT‐DC measurements. Emmanuel Manu performed AFM measurements. Ricardo H. Pires and Oliver Otto designed experiments, analyzed the data, and wrote the manuscript.

## Supporting information



Supplementary Material

Video S1

Video S2
