# Supplementary material for: Switching in the expression pattern of actin isoforms marks the onset of contractility and distinct mechanodynamic behavior during cardiomyocyte differentiation
Source: Physiol Rep. 2022 Feb 14;10(3):e15171. doi: 10.14814/phy2.15171 (PMC8844573; doi:10.14814/phy2.15171)
Supplement: Supplementary file 1 — Supplementary Material [file PHY2-10-e15171-s002.pdf]

SUPPLEMENTARY INFORMATION

Title

Switching in the expression pattern of actin isoforms marks the onset of contractility and distinct mechanodynamic behavior during cardiomyocyte differentiation

Running Title

Cell stiffness during cardiomyocyte differentiation.

Authors

Ricardo H. Pires[1,2] *, Tung H. Dau[1,3], Emmanuel Manu[1,2], Nithya Shree[1] and Oliver Otto[1,2] *

Affiliation

[1]ZIK-HIKE - Zentrum für Innovationskompetenz: Humorale Immunreaktionen bei kardiovaskulären Erkrankungen, Universität Greifswald, Fleischmannstr. 42, 17489 Greifswald, Germany

[2]DZHK - Deutsches Zentrum für Herzkreislaufforschung, Partner Site Greifswald, Fleischmannstr. 9, 17489 Greifswald.

[3]FLI - Friedrich-Loeffler-Institut, Südufer 10, 17493 Greifswald, Insel Riems, Germany.

Correspondence

* Ricardo H. Pires (piresr@uni-greifswald.de) and Oliver Otto (oliver.otto@uni-greifswald.de).

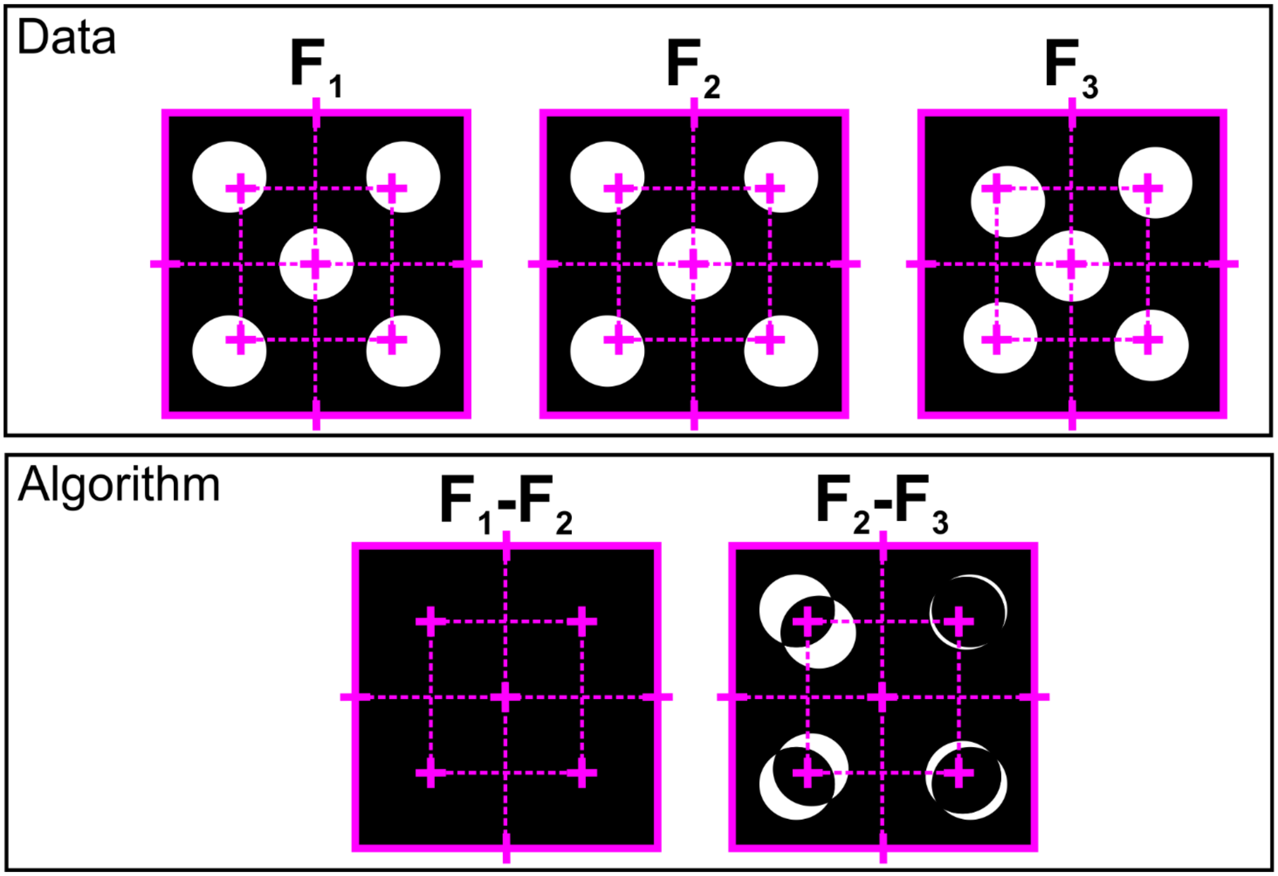

Supplemental Figure 1: Diagram illustrating the concept of motion detection implemented in this work.

The algorithm is based on difference in pixel grayscale intensity between consecutive frames. (above) Five circular objects imaged in F_1 and F_2 do not change their position, but some move from F_2 to F_3. (below) The output of the algorithm shows an image with pixels with grayscale intensities with values close to zero in the absence of motion (F_1-F_2), and non-zero when displacements occur (F_2-F_3).

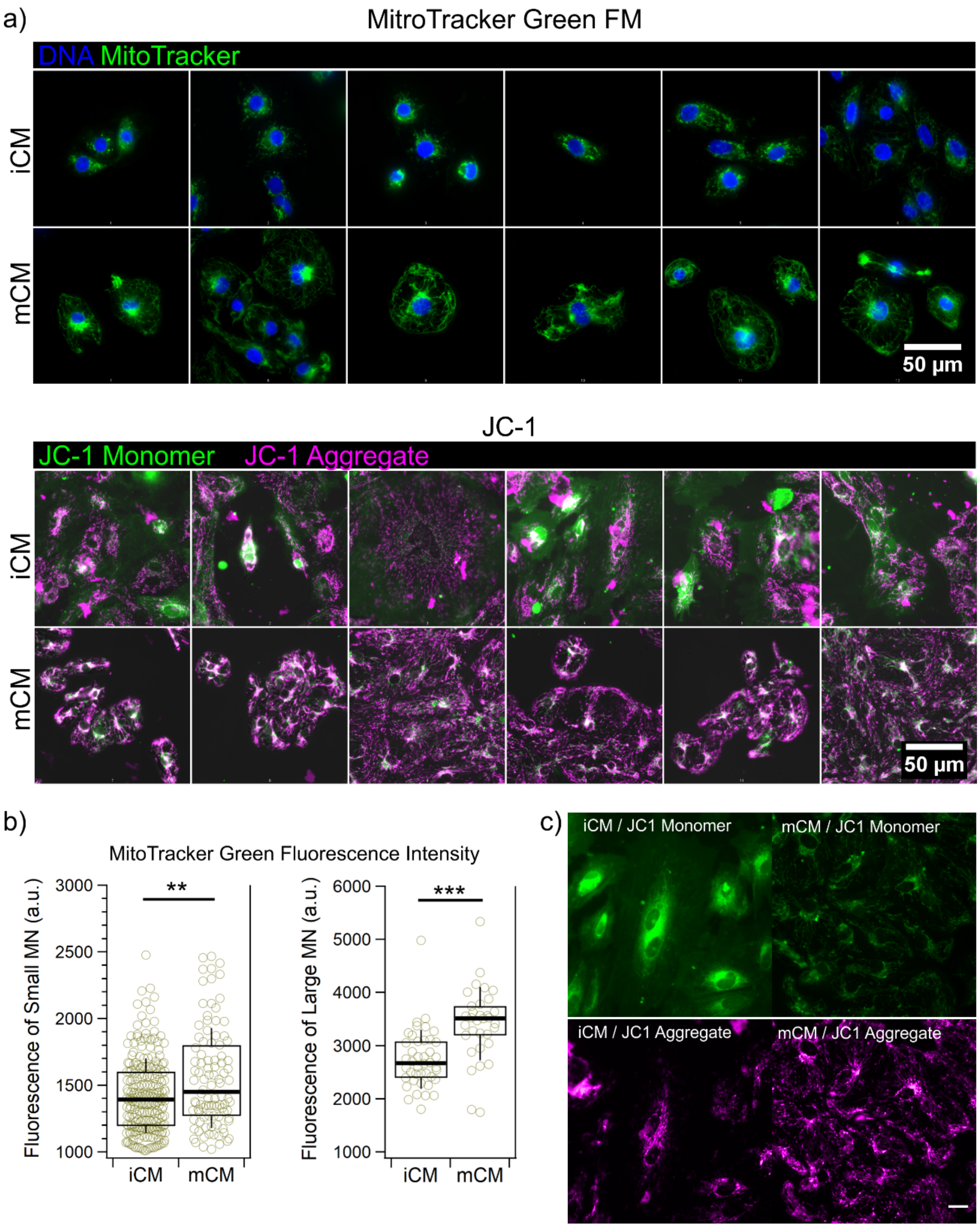

Supplemental Figure 2: Live cell fluorescence imaging of mitochondria.

- **a)** Gallery of cells imaged with Mitotracker Green FM (top) and composite images of cells labeled with JC-1 mitochondrial probe (bottom).
- **b)** Both large and small mitochondrial networks (MN) also show differences in mitotracker green fluorescence intensity between iCM and mCM possibly due to differences in their intracellular density.
- **c)** Single channel images of the composite image of JC-1 labeled cells shown in the main part of the manuscript in **Figure 2c**.

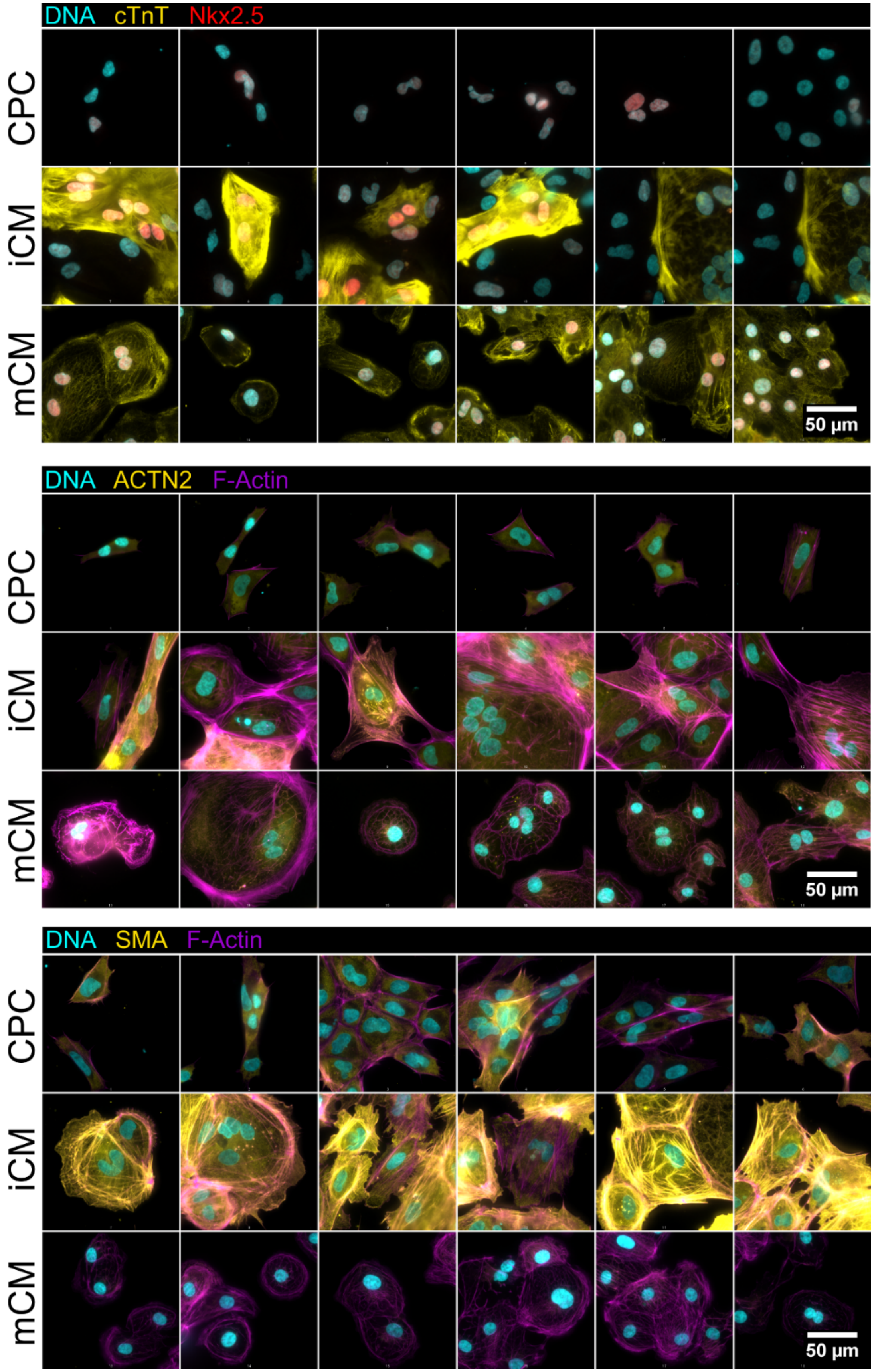

Supplemental Figure 3: Expression of cytoskeletal proteins. Gallery of images displaying the differences in the expression level of different proteins.

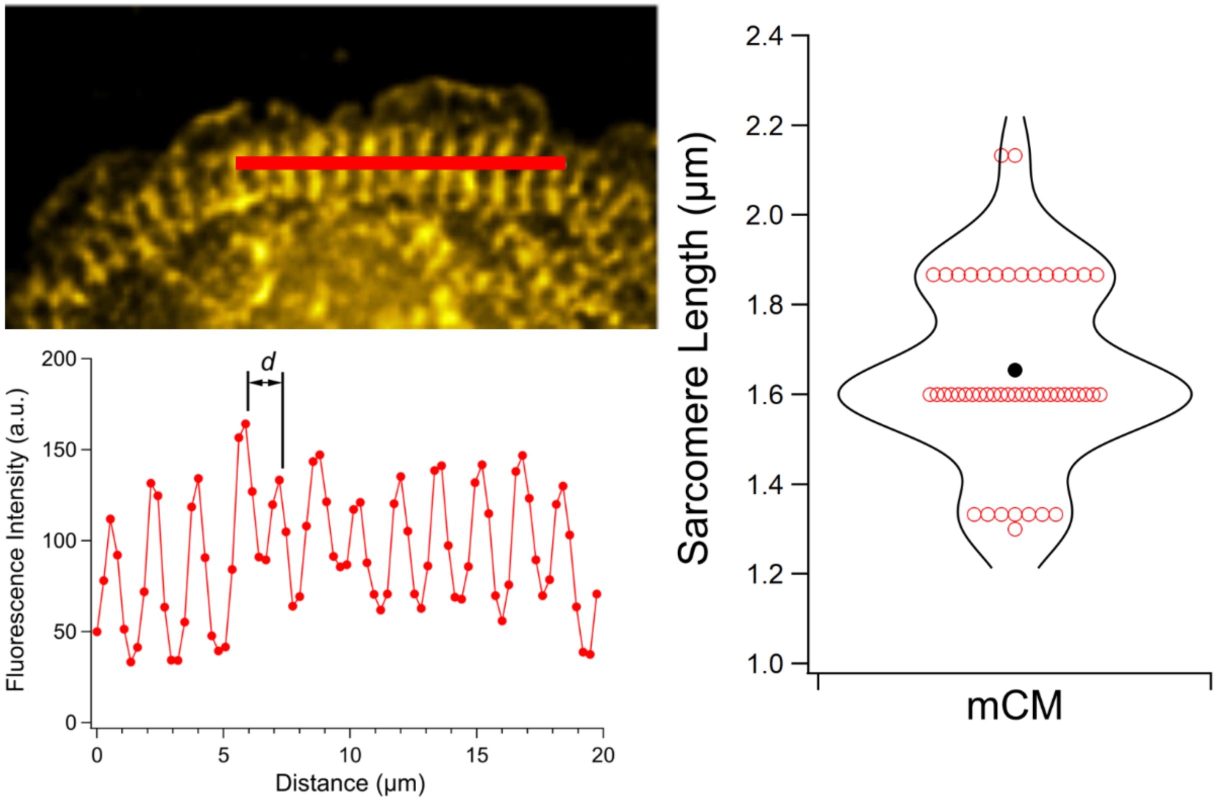

Supplemental Figure 4: Measurement of sarcomere length in mCM. A typical measurement of sarcomere length in mCM labeled with anti-cardiac sarcomeric α-actinin (top left) results in a corrugated fluorescence intensity profile (bottom left). Measurements of distance (*d*) between peaks is indicative of the distance between Z-disks, or the unit length of a sarcomere, which in the case of mCM is 1.65 ± 0.03 µm (mean ± s.e.m, n = 49) and ranged between 1.3 and 2.1 µm (right).

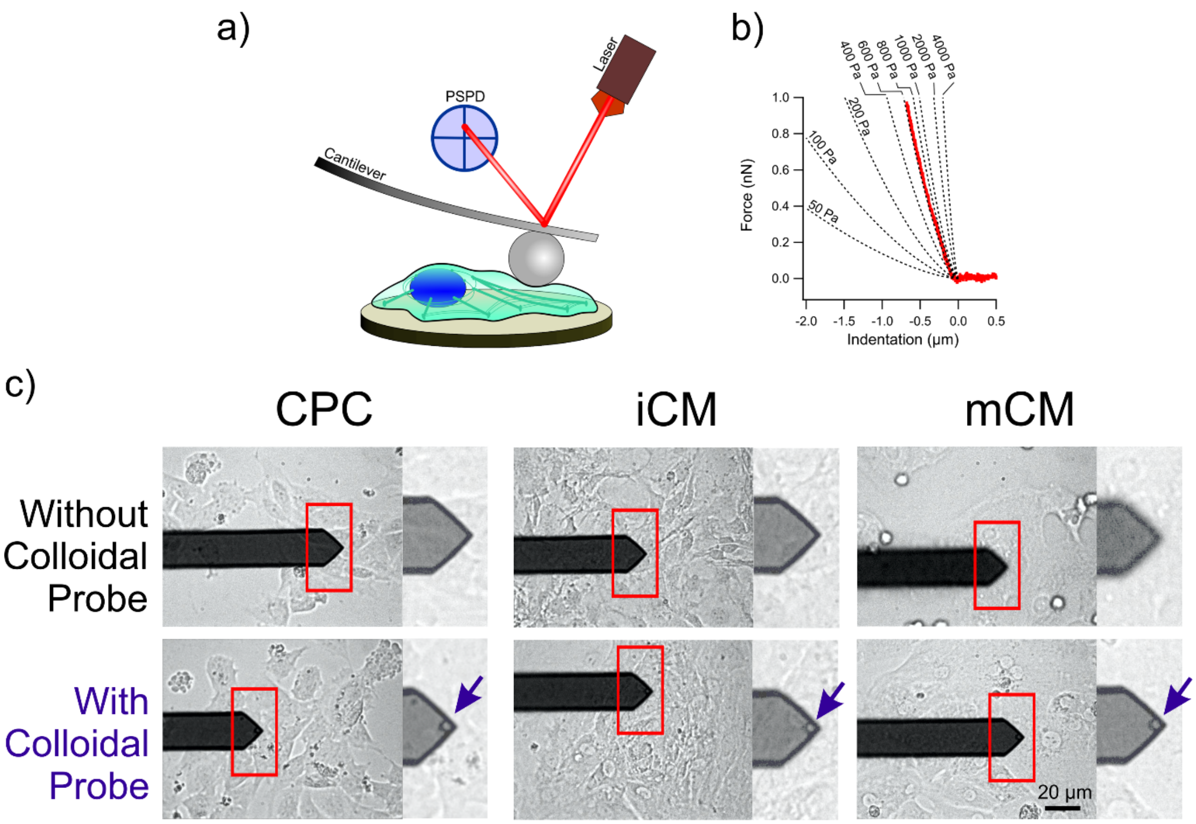

Supplemental Figure 5: **AFM colloidal force spectroscopy indentation experiments.**

(a) Cells are pressed with a spherical indenter placed at the extremity of a flexible cantilever. Bending of the cantilever as it presses on the sample is monitored via a laser deflected from the back of the cantilever onto a position sensitive photodiode (PSPD). Calibration of the cantilever ensures that bending can be expressed as indentation force.

(b) Example of a typical single indentation event on a cell (red) graphed along isoelasticity lines (dashed) derived from the Hertz contact model (see Methods) for different Young's moduli, from 50 Pa up to 4000 Pa.

(c) Indentation experiments were performed with a colloidal probe whose presence (arrows) could be monitored during data acquisition.

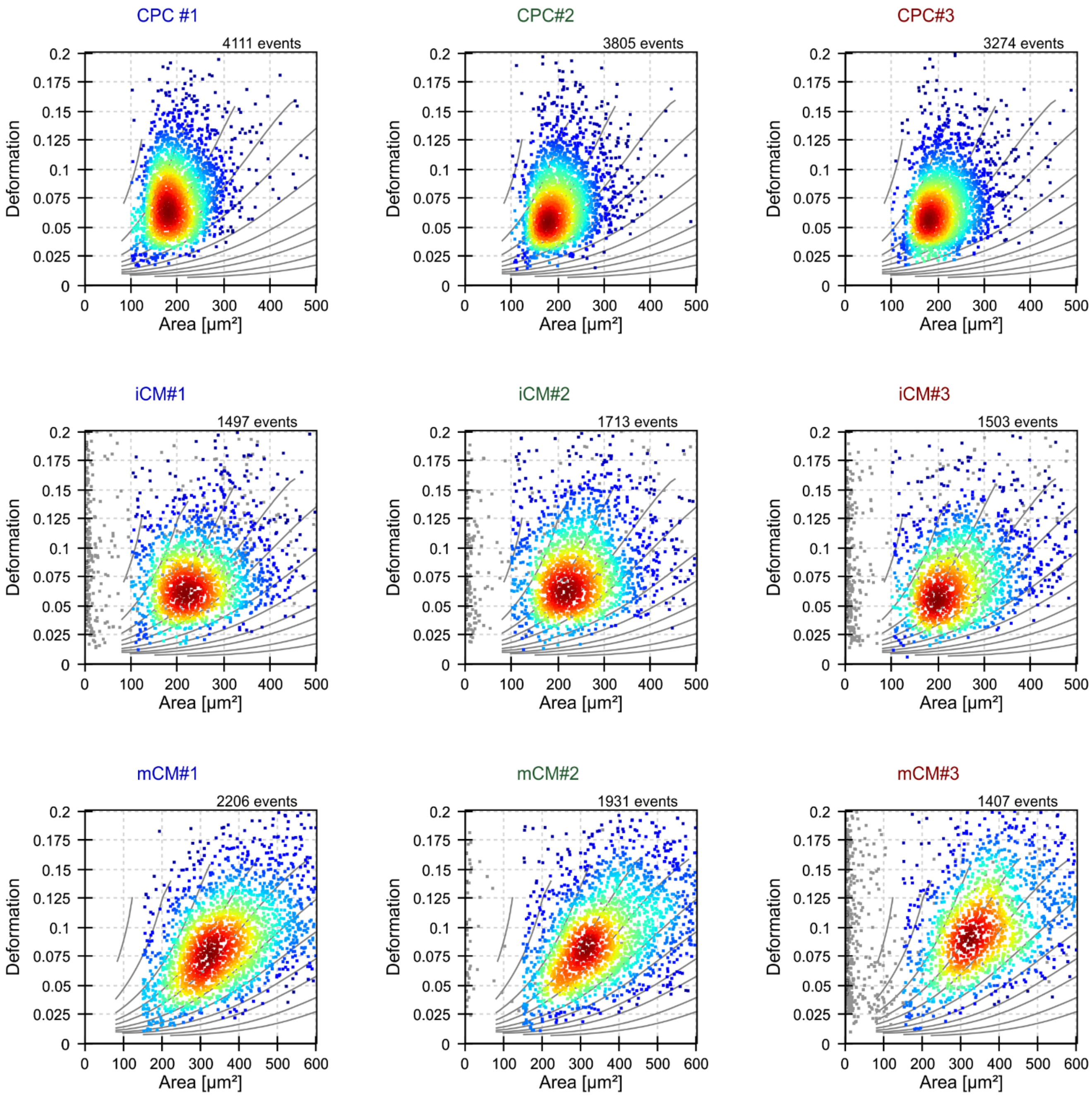

Supplemental Figure 6: Data plots obtained from real-time deformability cytometry (RT-DC) measurements.

Cell size (area) and cell deformation are measured in real-time through image analysis and the data is used to calculate the Young's modulus of cells shown as dots in the scatter-plots.

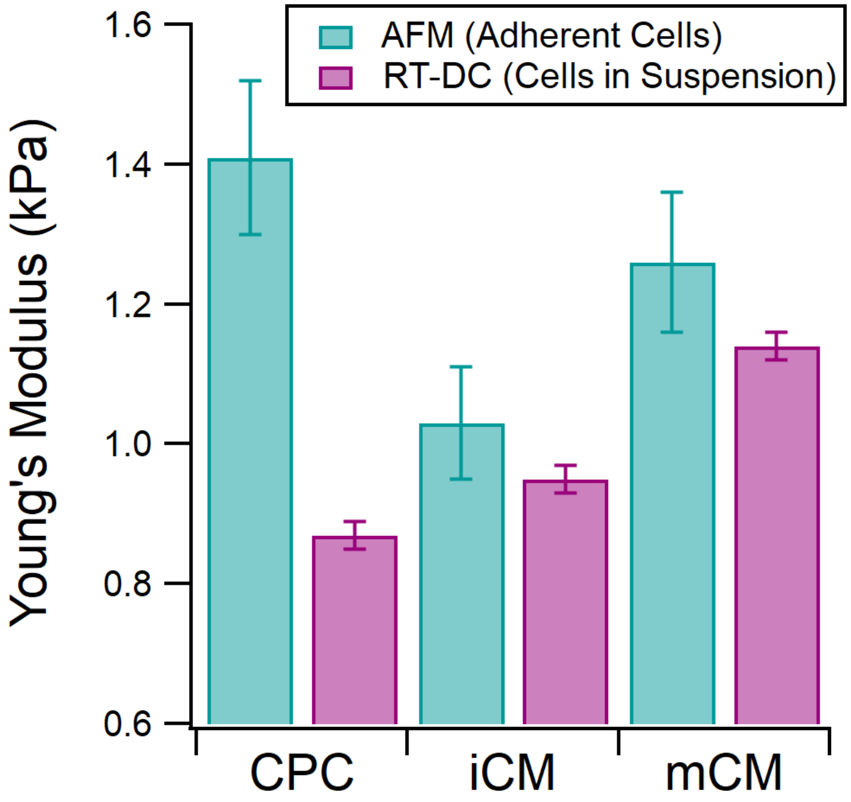

Supplemental Figure 7: Side-by-side comparison of the Young's modulus determined by AFM and RT-DC for cardiac progenitor cells (CPC), immature cardiomyocyte (iCM) and more differentiated cardiomyocytes (mCM).

Supplemental Table 1. Summary of AFM colloidal force spectroscopy indentation data.

Complete data was collected on two independent experiment replicates (A and B) for each of the cell type for any of the three conditions: negative control (control), vehicle control (DMSO) and 1 µM Cytochalasin D (CytoD). Data was filtered based on the fit residual root mean square (< 25 pN) and deviations in contact point between model and data (< 100 nm) resulting in an inclusion rate (Inc. Rate) that varied between 0.32 and 0.90. For each replicate, a total number (n) of force-indentations curves were used to calculate the mean Young's modulus (Mean YM) and associated standard error of the mean (s.e.m.).

Cell Type	Condition	Replicate	n	Inc. Rate	Mean YM (Pa)	s.e.m. (Pa)
**CPC**	Control	A	101	0.49	1600	161
**CPC**	Control	B	107	0.57	2431	177
**CPC**	DMSO	A	116	0.59	2482	212
**CPC**	DMSO	B	146	0.67	2152	152
**CPC**	CytoD	A	64	0.37	662	70
**CPC**	CytoD	B	51	0.40	714	224
**iCM**	Control	A	185	0.76	1831	453
**iCM**	Control	B	132	0.54	1682	159
**iCM**	DMSO	A	65	0.45	2345	280
**iCM**	DMSO	B	133	0.60	2299	176
**iCM**	CytoD	A	97	0.44	307	17
**iCM**	CytoD	B	102	0.53	737	157
**mCM**	Control	A	71	0.32	1534	123
**mCM**	Control	B	147	0.80	2212	232
**mCM**	DMSO	A	243	0.90	2107	223
**mCM**	DMSO	B	175	0.69	1854	134
**mCM**	CytoD	A	173	0.82	1671	193
**mCM**	CytoD	B	159	0.80	2242	204
